# Nano-Microparticle Platforms in Developing Next-Generation Vaccines

**DOI:** 10.3390/vaccines9060606

**Published:** 2021-06-05

**Authors:** Giuseppe Cappellano, Hugo Abreu, Chiara Casale, Umberto Dianzani, Annalisa Chiocchetti

**Affiliations:** 1 Dipartimento di Scienze della Salute, Interdisciplinary Research Center of Autoimmune Diseases—IRCAD, Università del Piemonte Orientale, 28100 Novara, Italy; giuseppe.cappellano@med.uniupo.it (G.C.); hugo.abreu@uniupo.it (H.A.); chiara.casale@uniupo.it (C.C.); annalisa.chiocchetti@med.uniupo.it (A.C.); 2 Center for Translational Research on Autoimmune and Allergic Disease—CAAD, Università del Piemonte Orientale, 28100 Novara, Italy; 3 Laboratorio di Biochimica Clinica, Dipartimento di Scienze della Salute, AOU Maggiore della Carità, Università del Piemonte Orientale, Corso Mazzini 18, 28100 Novara, Italy

**Keywords:** advanced vaccines, PLGA, liposome, extracellular vesicles, virus infection

## Abstract

The first vaccines ever made were based on live-attenuated or inactivated pathogens, either whole cells or fragments. Although these vaccines required the co-administration of antigens with adjuvants to induce a strong humoral response, they could only elicit a poor CD8^+^ T-cell response. In contrast, next-generation nano/microparticle-based vaccines offer several advantages over traditional ones because they can induce a more potent CD8^+^ T-cell response and, at the same time, are ideal carriers for proteins, adjuvants, and nucleic acids. The fact that these nanocarriers can be loaded with molecules able to modulate the immune response by inducing different effector functions and regulatory activities makes them ideal tools for inverse vaccination, whose goal is to shut down the immune response in autoimmune diseases. Poly (lactic-co-glycolic acid) (PLGA) and liposomes are biocompatible materials approved by the Food and Drug Administration (FDA) for clinical use and are, therefore, suitable for nanoparticle-based vaccines. Recently, another candidate platform for innovative vaccines based on extracellular vesicles (EVs) has been shown to efficiently co-deliver antigens and adjuvants. This review will discuss the potential use of PLGA-NPs, liposomes, and EVs as carriers of peptides, adjuvants, mRNA, and DNA for the development of next-generation vaccines against endemic and emerging viruses in light of the recent COVID-19 pandemic.

## 1. Introduction

Vertebrates have developed defense mechanisms consisting of innate and adaptive immunity [1] that collaborate to build an effective immune response against microbial invaders. Innate immunity is an ancient, fast, unspecific, local, and antigen-independent process, which is stimulated when membrane-associated or cytosolic pattern recognition receptors (PRRs) expressed on immune cells [i.e., dendritic cells (DCs), monocytes, macrophages, neutrophils, natural killer (NK) cells] recognize pathogen-associated molecular patterns (PAMPs) widely expressed in microbes [2]. Upon PAMP recognition, the host defense mechanism is activated, resulting in acute inflammation, crucial to recruiting immune cells to the site of infection [3], and activate adaptive immunity.

During viral infection, cellular immune responses mediated by CD4^+^ T helper (T_H_) cells and CD8^+^ cytotoxic T lymphocytes (CTLs) are crucial for host defense. T_H_ cells include T_H_1 cells, which potentiate phagocyte and NK cell cytotoxicity by secreting IL-2 and IFN-γ, and T_H_2, which secrete IL-4, IL-5, and IL-6, thereby enhancing antibody production by B lymphocytes [4,5]. CTLs recognize virus-infected cells and induce their apoptosis in order to clear the invading pathogens [6]. Besides eliciting an effector response that eradicates the infection, these processes contribute to the development of an immunological memory that can trigger an effective response when the same pathogen is encountered a second time [7]. Thus, by exposing the body to harmless forms of the pathogen, vaccines can build up an immunological memory in the absence of a true pathological infection [8]. This review will initially focus on first-, second-, and third-generation vaccines and will then address next-generation vaccines (graphical abstract).

## 2. Classical Vaccines

Classical vaccines were invented by Louis Pasteur in the late 1800s, following from the initial observation made by Edward Jenner about one century earlier that cowpox infection induced an immune condition that protected from smallpox infection, devising a new procedure nowadays known as vaccination—a term derived from the Latin word for cow “vacca” [9]. Later on, Pasteur succeeded in developing several other vaccines by isolating and inactivating disease-specific pathogens through different methods, de facto revolutionizing the biomedical field [10]. His work inspired classical vaccination techniques based on the use of whole microbes, which were either live attenuated (LA) or inactivated/killed [11].

LA vaccines (LAVs) are indeed developed from weakened pathogens that are able to proliferate in the host, causing mild disease or none at all. The immune response elicited by LAVs is similar to that induced by virulent pathogens and often confers long-term immunity in a single dose, without the need of adjuvants [12]. The main risk associated with LAVs is the reversion to virulence of the pathogen, which may lead to severe infection in immunocompromised individuals and may harm the fetus during pregnancy. In addition, LAVs generally need a temperature-controlled supply chain to preserve the living vaccine [13]. Despite these limitations, current LAVs for measles, rotavirus, and yellow fever, as well as the oral polio vaccine, are considered safe and suited for commercialization [11,13].

Inactivated vaccines consist of pathogens killed by chemical treatments. They are safer and more stable than LAVs and cannot revert to virulence or induce infection even in immunocompromised individuals. However, being less effective than LAVs in inducing immunity, they usually require several doses to generate a humoral response, often not permanent. Current examples are vaccines against poliomyelitis (i.e., inactivated polio vaccine—IPV), hepatitis A, rabies, and influenza [11,13].

Other types of vaccines are derived from antigenic subunits of the pathogen, generally polysaccharides or proteins [11]. Even though they are safe and stable, they can mainly induce a humoral response and thus require a careful choice of the antigen, which must be immunogenic enough to induce protective immunity against the target pathogen. [13,14]. Among this category, we find toxoid vaccines, which can inactivate tetanus or diphtheria exotoxins, and sub-viral particle-based vaccines, which can inhibit hepatitis B viral entry [11,13,14]. A limitation of these protein-based vaccines is that the denaturation and renaturation steps required for their production may alter the exposed epitope, thereby affecting its immunogenicity [8,13,14]. Moreover, the proteins may be degraded by proteases before antigen recognition [14].

The creation of protein-based vaccines has been greatly improved by the use of recombinant DNA technology combined with nano/microparticle delivery systems. Examples include vaccines for human papillomavirus (HPV), containing two or four copies of the capsid protein L1, and hepatitis B, containing the hepatitis B virus surface antigen (HBsAg). These vaccines are widely used and confer protection with rare side effects [8,13]. Viral proteins can also multimerize into virus-like particles (VLPs) that can be more effectively recognized by the immune system upon vaccination [15,16].

To correct the poor immunogenicity of inactivated and subunit-based vaccines, they are usually co-administered with adjuvants. These compounds significantly increase the vaccine immunogenicity by acting as both immunopotentiators and delivery systems [14,17]—described later in this review. The most common adjuvant is aluminum, in the form of several salts—e.g., aluminum phosphate, aluminum hydroxide, and aluminum potassium sulfate (Alum)—which has been administered with diphtheria vaccines since the 1930s [18,19,20]. Alum promotes the recruitment of antigen presenting cells (APCs), thereby increasing their antigen uptake and presentation. Furthermore, it induces APC maturation and migration to the draining lymph node, and it stimulates a T_H_2 response, supporting antibody production [19]. Aluminum compounds combined with monophosphoryl lipid A (MPLA) have also been used to create a vaccine against human papillomavirus (HPV). Other types of adjuvants include emulsions, such as MF59, an adjuvant composed by squalene in citric acid buffer found in influenza vaccines [21], and DNA sequences, such as the CpG 1018 oligonucleotide, a Toll-like receptor 9 (TLR) agonist adjuvant that, when co-administered with HBsAg, boosts immunity against hepatitis B [22].

## 3. Nucleic Acid Vaccines

Upon injection in a tissue, exogenous nucleic acids (DNA or RNA) can be captured by cells and eventually translated into protein(s), which are then released or presented to immune cells [23]. DNA vaccines are usually developed by incorporating eukaryotic DNA constructs into bacteria-derived or semi/fully synthetic plasmids [23]. Importantly, plasmids never replicate in mammalian hosts, nor do they integrate in genomic DNA [24]. DNA vaccines are easy and fast to produce, are highly stable, and present no risk of infection since no live pathogen is injected [23,25]. Additionally, they induce both cell-mediated and humoral immune responses since the plasmid can enter not only structural tissue cells, such as myocytes, but also APCs, capable of presenting the vaccine antigens through MHC class I and II molecules. Therefore, these vaccines can induce a complete immune response involving CD4^+^ T helper cells—T_H_1 and T_H_2—CD8^+^ cytotoxic T cells, and B cells [23,26]. Even though studies performed in different species demonstrated a safe and non-integrative profile of DNA vaccines [25,27,28,29], these concerns have cast doubt on their safety so that, to date, they have yet to be approved for use in humans, whereas four of them have already been licensed for veterinary use [25]. Recently, several clinical trials of DNA vaccines for Ebola, Zika, and influenza H5N1 viruses have reported promising results regarding safety and efficacy, opening new avenues for their future use against viral diseases [23].

RNA vaccines can be made with non-replicating mRNA or self-replicating mRNA [23,30,31]. Non-replicating mRNA contains the sequence of the antigen of interest flanked by two untranslated regions (UTRs) at both the 5′ and 3′ ends of the sequence. It results from a linearized DNA plasmid transcribed in an in vitro system (e.g., *E. coli*) by a DNA-dependent RNA polymerase, usually derived from the T3, T7, or Sp6 phage. After purification, this process leads to a fully mature mRNA similar to that of typical eukaryotic transcripts, including a 5′ cap and a poly(A) tail for stability and translation enhancement [23,30]. Remarkably, this technique has allowed for the fast development of vaccines against SARS-CoV-2 that have been emergency-approved for mass immunization during the COVID-19 pandemic—further discussed in Section 5 [31].

Self-replicating or self-amplifying mRNAs (saRNAs) are constructs based on the alphavirus genome. These constructs retain the replication machinery of alphavirus thanks to the genes encoding the nsP1-4 complex, which assembles into an RNA-dependent RNA polymerase, whereas the genes encoding the alphavirus structural proteins are replaced by the mRNA encoding the target antigen. After entering the host cells, this mRNA construct can amplify itself, leading to high levels of antigen production and to a potentially robust immune response, thereby reducing the need of vaccine recalls [23,30,32]. Preclinical and clinical studies using saRNA vaccines for influenza, AIDS, rabies, and SARS-CoV-2 are underway—reviewed in [32].

In cases of large transcripts, it is possible to use a trans-amplifying approach based on the combination of two mRNA constructs: one encoding the alphavirus replication genes, and the other encoding the gene of interest. In this case, the nsP1-4 complex can also replicate the non-self-amplifying mRNA transcript with the same advantages as those afforded by self-amplifying mRNAs [32,33]. Tests performed in mice have shown that this approach can confer immunity against influenza upon the administration of two doses, 21 days apart [33].

Although DNA and RNA vaccines are cost-effective and associated with minimal side effect risks, they can be easily degraded upon delivery to the target sites [34,35].

## 4. Next-Generation Vaccines Based on Nano/Microparticle Delivery Systems

**Figure 1:**
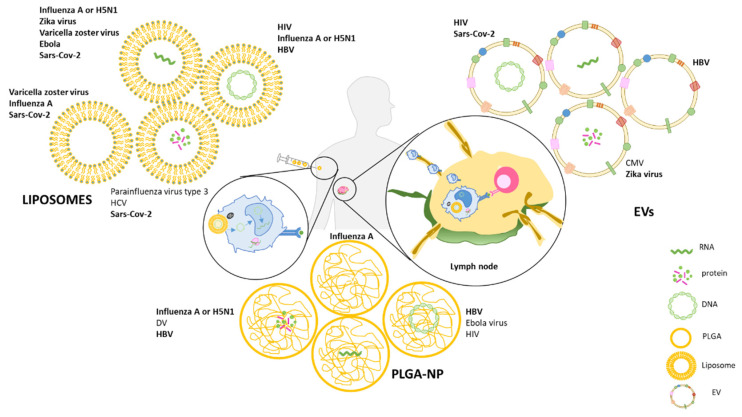
Overview of liposomes, EVs and PLGA-NPs as carriers for vaccines against viral infections. Proteins, plasmid DNA and mRNA have been successfully formulated in these NPs; empty NPs can also be used as adjuvant in vaccine formulation. Upon injection, they are internalized by APCs and, by reaching the lymph nodes, they present the viral antigen to T cells in order to induce an immune response. Some of these advanced vaccines were successful in eradicating the related viral infections (shown in bold). HBV: Hepatitis B Virus, HCV: Hepatitis C Virus; DV: Dengue Virus; CMV: cytomegalovirus; HIV: Human Immunodeficiency Virus: Sars-CoV-2: Severe Acute Respiratory Syndrome Coronavirus 2.

A further step of vaccine development is represented by engineered nanoparticles (NPs) used as vaccine delivery platforms that are able to protect the antigenic component of the vaccine while delivering innovative adjuvants that can finely tune the immune response. In addition to their delivery function, NPs display an intrinsic adjuvant activity, which makes them particularly suitable as vaccine platforms. Once internalized by APCs, NPs can in fact trigger inflammasome complex formation, which promotes the inflammation and recruitment of immune cells (Figure 1) [36,37,38]. Thus, NPs are promising antigen carriers and immune cell activators for the preparation of more effective vaccines. Of note is the fact that NPs can also be engineered to function as negative modulators of immune activation makes them attractive candidate for inverse vaccination—discussed in Section 4.2.5.

Several NPs are being tested to deliver protein- and nucleic acid-based vaccines. Among the most promising are biodegrading polymers in the form of poly (lactic-co-glycolic acid) NPs (PLGA-NPs), liposomes, and extracellular vesicles (EVs), which will be described in this review (Table 1).

### 4.1. PLGA

For several decades, poly(lactic-co-glycolic acid) (PLGA) has been used as a constituent of NPs because of its excellent biocompatibility, biodegradability, and safety profile [39]. Indeed, its use in humans was approved by the Food and Drug Administration (FDA) more than 30 years ago [40,41]. PLGA undergoes hydrolysis in the body to produce lactic acid and glycolic acid, which are efficiently metabolized through the Krebs cycle, thus avoiding toxicity [42].

PLGA-NPs have been validated as effective drug delivery systems by several studies in vivo. In particular, these compounds were shown to function as transporters of orally-administered insulin [43] and to be effective in delivering drugs to various body districts (e.g., cochlea, liver, and kidneys [44]), inflamed sites due to inflammatory diseases (e.g., arthritis [45] and Bowel disease [46]), and neoplastic tissues. They were also employed to produce tolerogenic vaccines for autoimmune diseases, such as experimental autoimmune encephalomyelitis (EAE), an animal model of multiple sclerosis [47,48]. In particular, PLGA was shown to be an excellent biocompatible polymer for NPs because PLGA-NPs could be loaded with a wide variety of molecules and their surface functionalized in order to improve their delivery to target tissues. In this regard, it is important to point out that vaccine delivery is highly influenced by the size of the NP. Indeed, small NPs elicit stronger humoral and cellular immune responses because they can more easily reach the lymph nodes and are more efficiently captured by APCs [49].

#### 4.1.1. Protein—Based PLGA Viral Vaccines

Influenza A virus, characterized by a high mutation rate, is known to cause seasonal infection waves worldwide [50]. Vaccines for influenza A induce the production of antibodies against the surface glycoproteins hemagglutinin (HA) and neuraminidase (NA) and must, therefore, be reformulated every year in order to be adapted to the antigenic drift of the virus. In this regard, a PLGA-NP-based vaccine for the H1N1 strain of influenza was obtained by loading NPs with the HA protein, together with MLPA and muramyl dipeptide (MDP), used as adjuvants that are capable of triggering a PRR-mediated response. This vaccine was shown to induce IFN-γ-producing CD4^+^ T cells and a strong antibody response. Interestingly, mice immunized with HA-PLGA-NPs plus the adjuvants were significantly more resistant to the lethal challenge with H1N1 virus compared to mice immunized without adjuvants [51].

Dengue virus (DENV) causes hemorrhagic fever and shock syndrome and represents a public health threat in Southeast Asia and Central and South America. A Dengue vaccine consisting of PLGA/PEG NPs loaded with viral nonstructural protein 1 (NS1) in the absence of adjuvants was shown to be effective in mice [52]. Subsequently, Metz et al. tested the efficacy of immunization with a tetravalent recombinant envelope (rE) protein subunit vaccine adsorbed into the PLGA-NP surface. This strategy led to a uniform antibody response against all four DENV serotypes, compared to the sole use of soluble antigens, demonstrating the promising potential of this approach for vaccine development [53].

A different strategy came from studies by Zhu et al. on immunization against hepatitis B virus. In order to promote a continuous release of HBsAg, the protein was loaded into PLGA-NPs. In addition, to increase the antigen uptake by APCs, the NP surface was functionalized by mannosylation so as to target mannose receptors. Mannose-grafted PLGA-NPs loaded with HBsAg induced successful antigen presentation, CD8^+^ T cell response, and secretion of IFN-γ and IL-2 [54]. In another study, HBsAg entrapped in PLGA-NPs positively charged with cationic particles (i.e., stearyl amine and polyethyleneimine) was given as aerosol to female Sprague Dawley rats to reach the lungs. This vaccination induced the production of antigen-specific IgG in the serum and IgA in the oral, vaginal, and bronchoalveolar lavages. The respiratory route of administration also induced a cell-mediated immune response, triggering the production of IFN-γ and IL-2 [55]. In another study, PLGA-NPs were loaded with hepatitis B core antigen (HBcAg), with or without MPLA. The results showed that the vaccine containing MPLA was highly effective at inducing a strong HBcAg-specific T_H_1 immune response [56].

PLGA-NPs can also be used for the delivery of poor soluble proteins. Roopngam et al. encapsulated the insoluble form of E2 envelope glycoprotein subtype 1b of hepatitis C virus (HCV1b-E2) in PLGA microspheres, showing that its continuous release from these microspheres induced a strong CD8^+^ T-cell immune response, as well as IFN-γ secretion in vaccinated mice [57]. Lastly, PLGA NPs have been used to deliver a multi-epitope vaccine against human T-cell leukemia/lymphoma virus type 1 (HTLV-1), an oncogenic RNA virus responsible for T-cell leukemia. Specifically, a multi-epitope chimera, consisting of the Tax, env, and gag immunodominant HTLV-1 epitopes, was encapsulated in PLGA-NPs together with the CpG-oligonucleotide adjuvant. The results showed that the vaccine induced a strong humoral response, and that NP encapsulation was crucial to improve antigen presentation and induce a strong cellular and mucosal immune response [58].

#### 4.1.2. PLGA in DNA Vaccines

Despite the advantages of DNA vaccines, only a few studies have shown that naked-DNA vaccines can induce a robust immune response in humans. One study showed that a HBsAg DNA vaccine was safe and well tolerated in a cohort of 12 healthy hepatitis-naïve human volunteers, where it induced an adequate immune response leading to virus clearance [59]. Because DNA plasmids are susceptible to fast degradation by nucleases, various particle formulations have been employed to precisely deliver DNA vaccines to tissues and overcome this degradation. In this regard, PLGA-NPs represent an interesting approach since they seem to provide a continuous DNA release while inducing a strong T-cell response. Indeed, oral administration of a single dose of PLGA-NPs loaded with HBsAg-DNA induced a long-lasting antigen-specific antibody response in BALB/c mice. Moreover, an effective antigen specific CTL response was detected in the spleen and gut-associated lymphoid tissue upon in vitro re-stimulation with HBsAg [60].

Another potential application of this approach is against Ebola virus, which causes hemorrhagic fever and multiorgan failure. To date, no human vaccine for Ebola has been approved. A feasible method of vaccination proposed by Yang et al. consists in Ebola DNA vaccine coated on PLGA-poly- l-lysine/poly-γ-glutamic acid (PLGA-PLL/γPGA) NPs, which is capable of inducing a strong immune response in mice [61].

Finally, the development of PLGA microspheres loaded with complexes of DNA and polyethylenimine (PEI) holds great promise for the design of vaccines against human immunodeficiency virus type 1 (HIV-1), which has so far remained elusive to vaccination thanks to its ability to impair and evade the host immune system. This approach may lead to more effective vaccines because PEI protects DNA from degradation during encapsulation and, upon intramuscular injection, the microspheres can release intact and penetrative PEI/DNA complexes for several days. Indeed, this vaccine induced strong antibody and CTL responses to HIV in mice [62].

Altogether, these results show that PLGA-NPs can effectively transfer the DNA vaccine to DCs and stimulate efficient CD4^+^ and CD8^+^ T-cell immune responses.

#### 4.1.3. PLGA in mRNA Vaccines

PLGA-NPs have recently gained increasing attention as potential platforms to deliver mRNA vaccines because of their ability to escape from endosomes alongside their excellent biodegradability and biocompatibility profile [63]. However, the negative charge of PLGA severely impairs the mRNA incorporation efficacy, which might account for the poor success in developing these vaccines thus far [64]. However, promising results have been obtained with PLGA/PEI NPs, which were shown to deliver mRNA encoding for green fluorescent protein (GFP) to human monocyte-derived DCs in order to elicit the host immune response and eliminate any hypothetical pathogen [65].

#### 4.1.4. PLGA as Adjuvant in Vaccine Formulations

NPs have the potential to boost the immune response even without the encapsulated antigen. For instance, Seth et al., immediately before injection in BALB/c mice, mixed PLGA-NPs with a modular capsomere comprising the antigenic M2e peptide (CapM2e) of influenza A virus, obtaining higher levels of anti-M2e IgG1 compared to the control [66]. Similarly, Zhang et al. used PLGA as an adjuvant for influenza A immunization. These authors administered PLGA-NPs, loaded or not, with the TLR-7 agonist imiquimod (IMQ), to mice along with HA derived from an H5N1 influenza vaccine (A/Anhui/1/2005). The results showed the upregulation of the anti-HA antibody response in mice injected with HA adjuvanted with either empty PLGA-NPs or PLGA-NPs loaded with IMQ compared with control mice injected with HA alone. Furthermore, this formulation also increased IFN-γ production detected in splenocytes compared to that induced by control immunization with HA alone or HA plus Alum as adjuvant, in an ex-vivo setting [67].

#### 4.1.5. PLGA NP in Inverse Vaccination

Inverse vaccination is aimed at specifically inhibiting pathologic immune responses, such as those responsible for autoimmune and allergic diseases, by inducing peripheral tolerance. Tolerogenic vaccines aim to preserve the host immune defense while avoiding severe opportunistic infections [68] which may occur using immunosuppressive drugs. While conventional vaccines induce humoral and cellular effector immunity, tolerogenic vaccines inhibit existing pathogenic effector/memory T cells by inducing either their anergy/deletion or suppression through regulatory T cells (Tregs) capable of maintaining long-lasting immune tolerance. Tregs may derive from pre-existing Tregs or differentiated CD4^+^ naive T cells [69].

A promising approach of tolerogenic vaccination takes advantage of NPs to deliver both antigens and “tolerogenic adjuvants” to trigger suppressive responses [70]. In particular, our group investigated the tolerogenic effect of PLGA-NPs loaded with the myelin oligodendrocyte glycoprotein (MOG)_35–55_ autoantigen and recombinant interleukin-10 (r IL-10), used as a tolerogenic adjuvant, in experimental autoimmune encephalomyelitis (EAE). Results showed that this combination was effective in ameliorating EAE and reducing both demyelination and T_H_1 and T_H_17 responses [47]. Subsequent studies confirmed the efficacy of this approach in other autoimmune diseases, as reviewed in [48]. Interestingly, a very recent study has suggested the effectiveness of subcutaneous injection of MOG_35–55_ PEGylated-containing PLGA-NPs without tolerogenic adjuvants in ameliorating the EAE course [71]. Moreover, two other studies performed in mice have shown that oral vaccination with collagen II [72] or nasal vaccination with HSP70 [73]—in both cases, the antigens were incapsulated in PLGA-NPs—conferred a high level of protection against rheumatoid arthritis-like disease in the absence of inverse adjuvants.

### 4.2. Liposomes

Liposomes are spherical artificial vesicles derived from natural phospholipids and cholesterol [74] that, due to their excellent versatility and plasticity, are emerging as promising tools for vaccine development. Liposomes are safe and have already been successfully translated into clinical use [75]. In the context of vaccination, liposomes passively target their contents to APCs, diffuse into the lymph nodes, and ultimately enhance immune responses [76,77].

The research efforts on liposome-based vaccines have expanded enormously in the course of the last year in the attempt to develop effective vaccines against SARS-CoV-2. Thanks to the possibility to modulate liposome features (i.e., lipid composition, charge, and size), both hydrophilic and lipophilic molecules, such as proteins, peptides, nucleic acid, and adjuvants, can be entrapped within the liposome lipid layer or exposed on the liposome surface through chemical linking.

#### 4.2.1. Protein-Based Liposome Vaccines

Synthetic peptides are safe and can be prepared as pure immunogens in large quantities, which makes them ideal tools for vaccination. However, these peptides are weakly immunogenic and need the help of adjuvants to overcome this limitation. In this regard, liposomes are ideal to provide adjuvant activity as they induce innate immune responses and improve antigen delivery, which mounts robust adaptive immune responses. Moreover, peptide-vaccines can be equally effective with the peptides being either encapsulated into the liposomes or chemically coupled with the liposome surface. Liposome-coupled peptides are taken up by APCs through either direct fusion with the plasma membrane or pinocytosis [78]. Senchi et al. synthesized an oligomannose-coated liposome vaccine against human parainfluenza virus type 3 (HPIV3), which causes acute respiratory infections and asthma in children, by combining the HPIV3 hemagglutinin-neuraminidase antigen with the adjuvant poly(I:C). Intranasal administration of low doses of this vaccine protected mice from HPIV3 infection through induction of antigen-specific IgG and IgA [79].

Intradermal injection of a liposomal cationic adjuvant formulation (CAF09) and a mixture of peptides (pepmix) spanning the entire sequence of the HCV nonstructural protein 3 (NS3) induced a vigorous CD4^+^ T-cell response. Importantly, it induced immunity against subdominant T-cell epitopes that were not efficiently targeted by either vaccination with full-length recombinant rNS3 or even infection with HCV [80]. Likewise, subcutaneous immunization with HCV-derived antigenic peptides coupled with the liposome surface (Lip-603) promoted a robust CD8^+^ T cell-mediated anti-viral immunity. This response was more effective than that obtained using the same peptides emulsified in incomplete Freund’s adjuvant [81]. Moreover, Ohno et al. selected two peptides from SARS-CoV, the virus causing severe acute respiratory syndrome (SARS), as HLA-A*0201-restricted CTL epitopes and went on showing that, upon linkage to the liposomal surface (Lip-N223 and Lip-N227), they were highly effective in inducing peptide-specific CTLs in HLA-A*0201 transgenic mice [82].

#### 4.2.2. Liposomes in DNA Vaccines

Cationic liposomes can protect DNA from degradation and, by interacting with negatively charged cell membranes, can be taken up by APCs where they eventually disassemble, thereby favoring DNA plasmid entry into the nucleus.

Qiao et al. showed that, upon intramuscular immunization with mannosylated zwitterionic-based cationic liposomes (man-ZCL) decorated with an HIV DNA plasmid Env, mice developed T_H_1/T_H_2 mixed immune responses [83]. Rodriguez et al. used liposomes to deliver DNA plasmids encoding bovine herpesvirus type 1, demonstrating that immunized mice were able to develop specific IgG responses [84]. In another study, upon oral administration of a cationic liposome/DNA vaccine encoding the M1 gene of influenza A virus, immunized mice developed not only an M1-specific IgG antibody response but also antigen-specific CTLs [85]. Another group generated cationic liposomes loaded with a DNA vaccine encoding middle (pre-S2 plus S) envelope proteins of HBV together with the CpG-oligodeoxynucleotide adjuvant. This vaccine was successively transcutaneously injected into the mouse skin through microneedle to achieve a sustained release and long-lasting gene expression. Results showed that this transcutaneous immunization led to a balanced Th1/Th2 cell response [86]. Finally, cationic liposome-DNA complexes (CLDCs) were used as adjuvants for a vaccine containing an influenza H5N1 subunit and intramuscularly injected in mice. This immunization not only induced robust serum antibody and T_H_1/IFN-γ responses compared those observed in mice immunized with an unadjuvanted vaccine, but it also protected mice from influenza virus infection after just one jab [87].

#### 4.2.3. Liposomes in mRNA-Based Vaccines

mRNA-based vaccines loaded in liposomes represent a promising alternative to conventional vaccines to fight viral infection [30] and are known to induce anti-cancer immunity [88]. One of the advantages of using mRNA-liposome vaccines lies in their ability to elicit both humoral and cellular immunity, which are both required to eradicate intracellular pathogens, whereas subunit vaccines and killed/inactivated vaccines mainly elicit humoral immunity. This distinctive feature of mRNA-liposome vaccines is due to their ability to deliver the mRNA directly to the cytoplasm of DCs, where the exogenous mRNA is rapidly translated into the antigenic proteins that will then be processed by the proteasome to generate peptide epitopes to be presented to CD8^+^ T cells through MHC class I molecules [89]. Liposome mRNA-vaccines are regarded as safe since the exogenous mRNA, unlike DNA, cannot integrate into the host genome [90].

The pioneering study attempting to load mRNA in liposomes was published in 1978 by Dimitriadis et al. [91]. The authors entrapped rabbit globin mRNA sequences into liposomes and then successfully transfected them into mouse lymphocytes, thus providing the proof-of-concept of their approach. About fifteen years later, Martinon et al. demonstrated in vivo that injection of liposomes containing mRNA encoding the influenza virus nucleoprotein induced strong CTL responses in mice [92]. Another study showed that intranasal injection of liposome loaded with double-stranded RNA (LE-PolyICLC) was effective in eradicating influenza virus (H5N1-HPIV) by inhibiting virus replication, reducing viral titers, increasing survival of infected mice, and attenuating pulmonary fibrosis. Moreover, this compound displayed adjuvant activity when combined with an inactivated H5N1 vaccine, leading to enhanced humoral and cellular responses [93]. Others showed that liposome could be used also for passive immunization as intramuscular injection of an mRNA encoding ZIKV-117—a human anti-Zika neutralizing antibody—encapsulated in liposomes, conferred protection against Zika in mice [94]. Liposomes were also employed by Pardi et al. to develop an mRNA vaccine encoding influenza virus HA. Specifically, the authors demonstrated that immunization with HA mRNA-liposomes induced antibody responses against the HA stalk domain of influenza virus in mice, rabbits, and ferrets [95].

An additional advantage of mRNA vaccines is that they have been proven to be much more effective than LAVs. This was initially shown by Monslow et al. reporting that mRNA encoding the gE antigen of varicella-zoster virus (VZV) encapsulated in lipid NPs conferred a stronger immune response than that elicited by live attenuated VZV [96]. Liposomes have also been used to immunize guinea pigs with Ebola envelope (env) mRNA, leading to a strong response in terms of specific neutralizing IgG and 100% survival following Ebola virus infection [97]. Importantly, liposomes encapsulating the spike protein mRNA of SARS-CoV-2 are effective in inducing immunity against SARS-CoV-2. These vaccines are being currently administered worldwide and are under evaluation for the assessment of long protective responses [98]. A specific paragraph is dedicated to this topic in Section 5.

#### 4.2.4. Liposomes as Adjuvants in Vaccine Formulations

Liposomes are highly effective in overcoming the weak immunogenicity of subunit vaccines as they can carry adjuvants to further enhance the immune response. For instance, liposomes can be loaded with pathogen-derived molecules capable of triggering PPRs, such as TLRs or C-type lectin receptors (CLRs) [99]. In particular, Wui et al. developed a lyophilized vaccine, mixing cationic liposomes with the TLR4 agonist de-O-acylated lipooligosaccharide (dLOS), Quillaja saponin fraction QS-21, and the recombinant varicella zoster virus (VZV) glycoprotein E. This formulation was shown to induce a strong T_H_1 response in immunized mice [100]. Another study showed that the administration of trivalent influenza vaccine with the cationic liposome adjuvant system CAF01 enhanced both humoral and cellular immune responses in mice, followed by increased IL-1β, IL-2, IL-12, IFN-γ, and TNF-α [101]. Wørzne et al. reported that a single immunization of mice with the SARS-CoV-2 spike protein together with the adjuvant CAF01 significantly enhanced spike-specific CD4^+^ T_H_ responses, producing IFN-γ and IL-17, compared to other adjuvants, such as squalene emulsion (SE) and aluminum hydroxide. By contrast, the antibody responses against the spike receptor binding domain (RBD) was similar for all adjuvants [102]. Vaccines against hepatitis virus E mainly target the structural capsid protein open-reading-frame-2 (ORF-2) of the virus. Joshi et al. demonstrated that recombinant neutralizing epitope protein (rNEp), which is part of ORF-2, adjuvanted with liposomes, elicited a balanced T_H_1/T_H_2 response driven by DCs, while other adjuvants (i.e., Alum) induced a T_H_2 response driven by macrophages [103].

#### 4.2.5. Liposomes in Inverse Vaccination

Liposomes are not immunogenic per se, which makes them particularly suitable to inhibit autoimmune and allergic diseases through inverse vaccination, a process where vaccines are used to induce antigen-specific inhibition of autoimmune responses. In this regard, Kenison and colleagues found that a nanoliposome-based platform encapsulating the ligand of aryl hydrocarbon receptor and MOG_35–55_ suppressed EAE, in both prophylactic and therapeutic settings, by inducing several Treg cell types [104].

The rationale behind the use of liposomes for inverse vaccination is that DCs may acquire tolerogenic activity upon endocytosis of apoptotic cell material [105]. Thus, the fact that phosphatidyl-serine (PS) is exposed only on the apoptotic cell membrane [106] makes liposomes enriched in PS and loaded with autoantigens the ideal vectors to generate tolerogenic DCs with which to inhibit immune responses in autoimmune disease. For example, the intraperitoneal injection of PS-rich liposomes loaded with MOG_40–55_, before EAE onset, was shown to suppress EAE development and induce splenic forkhead box P3^+^ (foxp3) Tregs in mice [107]. The same strategy was also effective in non-obese diabetic mice, a model of type 1 diabetes, in which PS-liposomes loaded with insulin peptides induced tolerogenic DCs, impaired autoreactive T-cell proliferation, and inhibited the development of diabetes [108]. Furthermore, PS-enriched liposomes loaded with ovalbumin peptide 323 (OVA^323^) were shown to induce Tregs in wild-type mice that had received an adoptive transfer of splenocytes from OT-II mice, transgenic for an anti-OVA^323^ TCR, one day before immunization. Moreover, liposomes containing the anionic phospholipid 1,2-distearoyl-sn-glycero-3-phosphoglycerol induced Treg proliferation and reduced atherosclerotic plaque formation in apolipoprotein E (ApoE^−/−^) mice, a model of atherosclerosis [109]. Finally, in the murine OVA-induced model of allergic diarrhea, the treatment of sensitized mice with OVA loaded in oligomannose-coated liposomes induced regulatory CD8^+^ T cells, triggered IL-10 production, and ameliorated allergic diarrhea [110].

### 4.3. EVs as Delivery Systems in Vaccines

EVs are small lipid-based bilayer particles that are naturally secreted by almost all cell types [111]. Due to their size, origin, and content, they can be easily distinguished from exosomes, microvesicles, and apoptotic bodies. Both exosomes and microvesicles are physiologically involved in cell-to-cell communication and play a role in cancer cell-mediated modulation of the tumor microenvironment [112,113]. In addition, viral infection of mammalian cells can affect cellular EV content and secretion. For example, cytomegalovirus (CMV)-infected endothelial cells release viral antigen-containing EVs, which trigger CD4^+^ T cell activation [114]. Moreover, HIV-1 infection stimulates the release of T cell-derived EVs containing HIV Gag [115], an essential structural viral protein that contributes to the assembly, secretion, and maturation of HIV-1 [116].

EVs are natural carriers of several types of molecules, including nucleic acids (DNA and RNA), proteins and lipids, and have been shown to be safe, efficient, non-toxic, and weakly immunogenic carriers [112,117]. Furthermore, EVs can be engineered to express different surface markers, which can turn them into “antigen-presenting EVs”. Intriguingly, Tregs are able to release EVs with tolerogenic properties, which contributes to modulating the immune response without the need of direct cell-to-cell interaction. This activity could be either ascribed to the transfer of miRNAs or proteins to the target cells or attributed to the activity of surface proteins expressed on the vesicles [118]. Moreover, several studies performed in mice have shown that EVs derived from foxp3^+^ Tregs display suppressive functions mediated by molecules, such as CD73, which impair cytokine release from T cells by converting AMP into adenosine [119], or let-7d miRNA, which suppresses T_H_1 proliferation and IFN-γ release by inhibiting cyclooxygenase-2 (Cox-2) [120].

Altogether, the aforementioned features make EVs attractive candidates as delivery platforms in vaccine settings. Albeit pioneering studies have been focused on anti-cancer therapies, the attention has now shifted to viral disease. Of note, EVs are also used as biomarkers of several human diseases, including viral infections [121,122].

#### 4.3.1. Protein-Based EV Vaccines

Several types of engineered EVs containing viral proteins have been recently generated for immunization against viral infections. In particular, Admyre et al. treated monocyte-derived DCs with EVs loaded with 23 different peptide sequences (i.e., CEF peptide mix), originated from CMV, influenza, and Epstein-Barr virus, to test the immune response in vitro. The authors found that these EVs induced high levels of IFN-γ production from CD8^+^ T cells, which directly correlated with the number of EVs and expression levels of MHC class-I molecules [123].

More recently, Martins et al. fused bacterial EVs—also referred to as outer membrane vesicles (OMVs)—derived from *Neisseria meningitidis* with the envelope proteins of Zika virus in an attempt to create a vaccine against Zika infection. The immunization of mice with these particles induced an immune response, producing antibodies, IL-2 and IL-4. The authors proposed that the use of this innocuous and rapidly generated vehicle might be a promising approach for vaccine formulation [124].

#### 4.3.2. EVs in DNA-Based vaccines

Several efforts have been made to use EVs to produce valuable alternatives to conventional DNA vaccines. Di Bonito et al. showed that a DNA plasmid encoding a mutated HIV Nef protein (Nef^mut^), unable to downregulate CD4 and MHC class-I, fused with HPV E7 protein, induced the production of EVs expressing the protein chimera due to the membrane-anchoring properties of Nef. Intramuscular injection of this DNA plasmid induced a powerful CTL response against both Nef and E7, which was not achieved using wild-type Nef or E7 alone [125]. Subsequently, the authors replicated these results by fusing the mutant Nef protein with other viral proteins, including hepatitis C virus (HCV)-NS3, Ebola virus (EboV)-VP24, EboV-VP40, and EboV-NP, West Nile virus (WNV)-NS3, influenza (Flu)-NP, and Crimean–Congo hemorrhagic fever (CCHFV)-NP [126]. More recently, Polak et al. generated a prototype of EV-based anti-SARS-CoV-2 vaccine that combines both DNA and peptide-based techniques. Immunization with this vaccine required a primary immunization with a DNA vector inducing in vivo production of SARS-CoV-2 spike protein-expressing EVs and subsequent boost immunizations using EVs expressing the spike proteins produced in mammalian cells in vitro. This vaccine induced potent humoral and cellular responses in mice, without the need of adjuvants [127].

#### 4.3.3. EVs as Adjuvants in Vaccine Formulation

EVs can also be exploited as adjuvants regardless of their antigen expression. Their role as immunopotentiators has been explored by Jesus et al. using EVs isolated from LPS-activated THP-1 monocytes. Immunization of mice with these EVs mixed with either a solution of HBsAg or a suspension of HBsAg-loaded poly(ɛ-caprolactone)/chitosan NPs induced a stronger cell-mediated immune response, marked by IFN-γ production, even though the humoral response was comparable to that induced by vaccination in the absence of EVs [111].

### 4.4. Limitations of Nano/Microparticle-Based Vaccines

The knowledge of the biological behavior of NPs in terms of distribution in vivo, at both the organ and cellular level, is still lacking. We do not know whether exposure to NPs over long periods of time may affect the human body. Another concern is related to the use of NPs as adjuvants, which may result in chronic inflammatory reactions. Moreover, we have yet to fully explore the physicochemical properties of NPs. If on the one hand NPs might increase the uptake by APCs, on the other hand they may reach other organs/tissues potentially leading to adverse biological effects (i.e., apoptosis or necrosis). Finally, another important issue concerns the ability of NPs to aggregate, which may block the blood vessels in the host leading to thrombosis.

## 5. COVID-19 Vaccines Based on Nano/Microparticle Platforms

Since late 2019, the novel β-coronavirus-SARS-CoV-2 has spread across the globe causing the coronavirus disease 2019 (COVID-19) pandemic [128]. Coronaviruses are single-stranded enveloped RNA viruses with a tropism for the lower respiratory tract. Upon binding of the virus spike (S) glycoproteins expressed on the viral surface to angiotensin converting enzyme 2 (ACE2) receptor expressed on type II pneumocytes, SARS-CoV-2 infects the host cells and induces a severe inflammatory reaction, producing high levels of pro-inflammatory cytokines (“cytokine storm”), such as IL-1β, IL-2, IL-6, IFN-γ, and TNF-α which are responsible for severe tissue damage and thrombosis [129]. Clinical symptoms of COVID-19 range from asymptomatic infection to respiratory failure—requiring mechanical ventilation and intensive care unit (ICU) admission—acute respiratory distress syndrome (ARDS), sepsis, and multi-organ dysfunction syndrome (MODS) [130].

Immediately after the declaration of pandemic state by the World Health Organization (WHO) on 11 March 2020, and as soon as the SARS-CoV-2 sequence became available in March 2020, many research centers around the world started developing safe and effective vaccines against SARS-CoV-2 [131]. To date, a large number of different approaches have been proposed. Among the 198 vaccine candidates at the time of writing this review, 44 are in clinical trials, and 10 are in late-stage clinical development [132]. The first two vaccines approved by the European Medicines Agency (EMA) were the BNT162b2 (Pfizer-BioNTech) and mRNA-1273 (Moderna), two mRNA vaccines encapsulated in liposomes targeting the viral spike protein [131,133].

Both BNT162b2 and mRNA-1273 were able to induce the synthesis of the viral spike proteins in the host and induce an effective and protective immune response against SARS-CoV-2. They are administered in two-doses, 21 days apart for BNT162b2 and 28 days apart for mRNA-1273 [133]. Notably, Polack et al. has shown that the efficacy of BNT162b2 ranges from 89% to 100% after the second dose, eliciting a strong induction of T_H_1 and CD8^+^ T cells and neutralizing antibody response [131]. Similarly, recent data have reported a 94.1% efficacy of mRNA-1273 in preventing COVID-19 disease [133]. These vaccines together with more traditional ones based on recombinant adenoviruses carrying the SARS-CoV-2 spike genes [134,135] are playing a key role in battling the pandemic.

## 6. Conclusions

There is a great need for harmonization and simplification of the roadmap for the design and development of novel effective vaccines. The use of nanotechnological platforms in vaccine development holds great promise and will likely allow the generation of safe and affordable formulations for preventing multiple infections, possibly in a single shot. It is, however, mandatory to understand the mechanisms of NP entry into the cells and activation of the adaptive immune responses, including any related toxicity effects, especially inflammation.

In the future, we need to devise NPs that can specifically bind to target cells to ensure that only these carriers adhere to those cells. In addition, the increasing proportion of the human population requiring vaccination and the emerging threat of new viruses and drift variants have highlighted the need to develop affordable broad-spectrum vaccines. The data reviewed here indicate that nano/microparticles platforms will play a central role in achieving this goal. Finally, the effectiveness of these platforms in loading large amounts of adjuvants and modulating the immune response opens new avenues for their use in tolerogenic vaccination, which is expected to revolutionize the management of autoimmune diseases.

## Figures and Tables

**Table 1:** List of advanced vaccines delivered by nano- and microcarriers for the treatment of viral infection.

Antigen	Nano/Microparticle Platform	Disease	Animal/Human
Hemagglutinin (HA)	PLGA-NPs adjuvanted with MLPA and muramyl dipeptide (MDP)	Influenza A [51]	BALB/c and C57BL/6 mice
Nonstructural protein 1 (NS1)	PLGA/polyethylene glycol (PEG)-NPs	Dengue [52]	BALB/c mice
Hepatitis B surface antigen (HBsAg)	mannose-grafted PLGA-NPs	Hepatitis B virus [53]	Balb/c mice
Hepatitis B core antigen (HBcAg)	PLGA-NPs with monophospholipid A (MPLA)	chronic hepatitis B infection [53]	C57BL/6J mice
Insoluble form of E2 envelope glycoprotein subtype 1b of hepatitis C virus (HCV1b-E2)	PLGA microspheres	Hepatitis C virus (HCV) [57]	Balb/c mice
Plasmid DNA encoding HBsAg	PLGA-NPs	hepatitis B virus (HBV) [60]	Balb/c mice
M2e peptide (CapM2e)	PLGA-NPs	Influenza A [66]	Balb/c mice
Hemagglutinin-neuraminidase (HN)	oligomannose-coated liposome and Poly(I:C) as adjuvant	human parainfluenza virus type 3 (HPIV3) [79]	BALB/c mice
Mixture of peptides (pepmix) spanning the entire sequence of nonstructural protein 3 (NS3)	cationic liposomes (CAF09)	chronic hepatitis C virus (HCV) [80]	CB6F1 (C57BL/6 × BALB/c) and C3H mice
Four HLA-A*0201-restricted cytotoxic T lymphocytes (CTL) epitopes	Liposomes	Severe acute respiratory syndrome (SARS) coronavirus (SARS-CoV) [82]	HLA-A*0201 transgenic mice
Influenza virus nucleoprotein (NP)	Cholesterol/phosphatidylcholine/phosphatidylserine liposomes	Influenza virus [92]	Mice
Hemagglutinin (HA)	Lipid nanoparticles (LNPs)	Influenza virus [95]	Mice, rabbits, and ferrets
VZV gE antigen	LNPs	Varicella-zoster virus (VZV) [96]	Indian rhesus macaques
Ebola envelope glycoprotein	LNPs	Ebola virus [97]	Guinea pigs
SARS-CoV-2 spike protein	LNPs	severe acute respiratory syndrome coronavirus 2 (SARS-CoV-2) [98]	Healthy human adults
Recombinant VZV glycoprotein E (gE)	Cationic liposomes with the TLR4 agonist de-O-acylated	Varicella zoster virus (VZV) [100]	BALB/c and C57BL/6 mice
Trivalent influenza vaccine (TIV)	Cationic liposome adjuvant system CAF01	New influenza A (H1N1) [101]	BALB/c mice
Spike receptor binding domain (RBD)	Three different adjuvant systems: an aluminum hydroxide (AH), an oil-in-water squalene emulsion (SE) adjuvant resembling MF59™, a cationic liposome-based adjuvant (CAF^®^01)	Severe acute respiratory syndrome coronavirus 2 (SARS-CoV-2) [102]	C57Bl/6 mice
recombinant neutralizing epitope protein (rNEp), a part of the structural capsid protein open-reading-frame-2 (ORF-2)	Liposomes	Hepatitis E virus (HEV) [103]	Mice and rhesus macaques

## Data Availability

Not applicable.
